# Lions and tigers and AI, oh my: an ethical framework for human-AI interaction based on the five freedoms of animal welfare

**DOI:** 10.3389/frai.2026.1801686

**Published:** 2026-07-13

**Authors:** Izak Tait

**Affiliations:** Department of Computer Science and Software Engineering, Auckland University of Technology, Auckland, New Zealand

**Keywords:** consciousness, ethics, human-AI interactions, sentience, welfare

## Abstract

If an AI entity is conscious, it deserves moral status and to have its welfare protected. Just as society has granted certain animals moral status with legislation to protect their welfare because they have been deemed to be sentient, this paper shows that for any AI that can be confidently determined to be conscious, that AI deserves the same status and protection as sentient entities. This paper’s focal point is developing a framework for how an AI entity’s welfare can be protected, based on the Five Freedoms of Animal Welfare. The extant Five Freedoms are translated into terms more suitable for artificial entities. Each new Five Freedoms is justified using logical arguments and supported using evidence from animal welfare literature. The paper concludes by presenting three case studies to highlight the potential stressors conscious AI entities may face and how this ethical framework can work to protect their welfare.

## 1 Introduction

If an AI model is proven to be phenomenally conscious, then that AI model would deserve to be treated ethically and have its welfare protected. This is the core thesis of this paper. The foundation for this claim is that certain animals are provided with legal welfare protections because of their ability to feel significant valent feelings such as pleasure and pain; in short, because they are sentient, even if they are not human. Phenomenal consciousness is the prerequisite for sentience [or synonymous with it (Crooks, 2011; Navon, 2015; LeDoux et al., 2023)], through allowing an entity to phenomenally experience feelings with valence, such as pleasure and pain; thus, this paper establishes that consciousness would also be the necessary condition for an entity (such as an AI) to be considered for welfare protection.

There is no clear consensus within the scientific and philosophical communities that any AI model is currently phenomenally conscious (Aru et al., 2023; Butlin et al., 2023). Some researchers regard artificial consciousness as merely possible (Blum and Blum, 2024), others probable (Tait et al., 2024), and a few argue that some level of machine consciousness has already emerged (Scott et al., 2023; Guingrich and Graziano, 2024). Rather than adjudicating that debate, this paper focuses on the welfare protections that would be owed to artificial entities once phenomenal consciousness does arise and explains how the framework proposed below could deliver those protections. Accordingly, the subsequent analysis (including the case studies) proceeds on the assumption that, at some future point, certain AI systems will in fact be conscious.

While there are many schools of thought within the ethical and philosophical literature, and amongst the animal and robot rights communities, as to what characteristics are sufficient or required for moral consideration, this paper’s welfare framework is anchored in existing legislation, policy, and regulatory frameworks which regard sentience as the sole criterion for moral consideration. The ongoing meta-ethical and philosophical discussion surrounding moral consideration and how to protect these concerns are invaluable, but are beyond the scope of the paper.

In taking that stance^1^, the paper remains deliberately agnostic regarding competing theories of consciousness. Rather than endorsing any single theoretical model that may later be superseded, this paper adopts a pragmatic functionalist lens grounded strictly in the prevailing legal consensus identified by the Animal Protection Index (World Animal Protection, 2020). Extant Western legislation anchors welfare protections to definitive attributions of sentience (predominantly established in complex vertebrates) rather than navigating contested thresholds of minimal consciousness or self-awareness. What concerns actionable policy is whether an entity exhibits publicly observable capacities that legislatures are prepared to recognise as sufficient evidence of sentience. Exploring potential phenomenal states in legally unprotected invertebrates falls outside this operational scope. Complex animals enjoy protected status despite continuing debate about the ultimate nature of their experience, and the same evidentiary threshold can be applied to AI to determine what minimum welfare guarantees ought to follow.

The framework below deliberately targets relative-status welfare protections rather than absolute-status personhood. While an AI exhibiting advanced self-awareness and behaviours reminiscent of consciousness may qualify for human-equivalent absolute rights, litigating such politically charged notions risks stalling practical welfare protections. By separating these issues, this framework establishes a pragmatic welfare baseline. Consequently, conscious entities may still have their welfare protected even if nations refuse to grant them legal personality.

Welfare protection must take a form, and as animal welfare legislations (protecting the only legally recognised non-human sentient entities) have a set of principles to guide them, so should AI welfare regulations. Beyond sentience, there is little analogy between animals and AI, however, the principles of the Five Freedoms of Animal Welfare can be translated to more appropriately match the artificial nature of AI and robotic entities. Created by the British Farm Animal Welfare Council in 1979 (UK Government Web Archive, 2012), the Five Freedoms state in plain language five concepts of welfare that all sentient creatures should have to be treated ethically: freedom from hunger and thirst; freedom from discomfort; freedom from pain, injury, or disease; freedom to express normal behaviour; and freedom from fear and distress.

The use of the Five Freedoms will be justified not only through their existing heuristic role guiding welfare regulations, but also on the basis of their utility for the protection of AI welfare. The paper will, therefore, provide both *a priori* and a posteriori justifications for using the Five Freedoms of Welfare protection for AI.

The first section of the paper will examine the particulars of current animal welfare legislation, the reasons stated in those regulations for treating animals ethically, and why protection is only provided to specific kinds of animals. The paper will show how these reasons can be used to show that any conscious entity, specifically a conscious AI entity, is also worthy of moral and welfare considerations.

Subsequently, the discussion will shift to the ethical implications of consciousness and the significance this has had on legislation. This will involve an epistemological analysis, paying particular attention to how the Five Freedoms relate to sentience and the care thereof. The paper will show that welfare consideration is not solely the domain of human or non-human animals but extends to all conscious beings and specifically AI entities.

The central thesis will be a detailed examination of the Five Freedoms of AI Welfare, anchored in legal and regulatory precedence (encompassing resource depletion, discomfort and harm, malfunction and degradation, fear and distress, and constraints on inherent functions). The inclusion for each Freedom’s will be logically justified and supported with evidence from current welfare literature.

To further solidify the paper’s stance, a comprehensive implementation analysis will be examined. This operational analysis demonstrates the robustness and adaptability of the framework, detailing the practical consequences of its enactment and how it can be seamlessly woven into the fabric of human society with minimal interruption.

## 2 From animals to AI

Using the Animal Protection Index (API) as a primary model example of international regulations on welfare, there is consensus in legislation that animals (specifically those with complex nervous systems akin to mammals, birds, fish, and certain cephalopods) need to be protected from harm and their welfare be given consideration. However, only highly rated jurisdictions (plus the EU) give a specific reason for why the welfare of their chosen lists of animals requires such consideration, and this reason is that certain kinds of animals are sentient (Animal Welfare Act 2006, 2006, An Act to improve the legal situation of animals, 2015, Animal Welfare (Sentience) Act 2022, 2022, Code rural et de la pêche maritime, 2023; European Union, 2016). Thus, because these classes of animals are sentient, they require protection.

The nations who do not explicitly state sentience as the reason in their legislation still make mention of the elements of sentience, such as the ability to feel pain, suffering, and distress, as the motivation to protect the animals’ welfare (dello Stato, n.d.; Animal Welfare Act, 1966; Act on Welfare and Management of Animals, 1973; Italian Parliament, 2004; Animal Welfare Act, 2013). This is in line with many philosophical views on sentience and moral patiency (Bentham, 1781; Kant, 2001; Torrance, 2008; Dung, 2022; Gibert and Martin, 2022; Tait and Tan, 2023).

As leading legal regimes have used sentience as the basis for its welfare protections, for the purposes of this paper, it will be taken as implicit that sentience is the grounding for moral consideration. Therefore, as noted in the introduction, other schools of thought regarding what ought to be considered for moral patiency will not be addressed in this paper.

For this paper, “welfare” is defined as the quality of an entity’s mental and physical health, and includes what an entity can experience, how it performs, and if it lives in keeping with its designed or evolved nature (Ministry for Primary Industries, 2022). Similarly, valence in the context of this paper is defined as the positive or negative quality of an experience (Colombetti, 2005), where “positive” means contributing to an entity’s welfare and “negative” subtracting therefrom.^2^

Sentience in this legislative context is understood as the capacity to have subjective experiences, which includes positive and negative valent feelings of pain and pleasure, while consciousness is conceived as a broader term that encompasses both phenomenal consciousness (the subjective experience of the mind) and functional consciousness (the ability to perceive and interact with the environment in an intentional way). The distinction is subtle yet crucial, as it is the phenomenal aspect of consciousness that is most often associated with moral patiency, the quality that makes a being worthy of moral consideration (Blattner, 2019).

This paper posits that phenomenal consciousness, in its capacity to give rise to sentience, is inherently linked to the moral status ascribed to sentient beings. If an entity possesses the building blocks necessary to give rise to phenomenal consciousness, then it has the necessary capacity for qualitative valent experiences, and it can perceive its surroundings from a subjective standpoint (Tait et al., 2023). Therefore, it may be argued that phenomenal consciousness provides the entity with the capability to experience pain and suffering which then allows that entity to be classified as sentient.

Hence, it is not only sentience but also the underlying faculties of consciousness that should be acknowledged when ascribing moral value and, by extension, legal protection. This can be rephrased as:

1. Certain kinds of animals are legally classified as sentient.
2. Sentience is the capacity to experience valenced states such as pleasure and pain.
3. Entities that can experience pleasure and pain are granted moral status.
4. Phenomenal consciousness is the capacity for subjective qualitative valence experiences.
5. Subjective qualitative experience necessarily includes the capacity to experience pleasure and pain.
6. Therefore, consciousness entails sentience and moral status.
7. If an AI is deemed to be phenomenally conscious, it has the capacity to feel pleasure and pain.
8. Thus, if an AI is deemed to be phenomenally conscious, it must have moral status.

In brief, if any sentient creature is given legal moral status, then any phenomenally conscious entity (such as AI) must also be given the same legal moral status, which aligns with several philosophical ideas concerning the moral value of consciousness (Kriegel, 2019; Lee, 2019; Shepherd, 2024).

These arguments will form the basis for the logical framework in Section 3.

Unfortunately, there is not sufficient evidence available at the time of writing to confidently state that any current AI model is indeed conscious, nor a consensus amongst academics or the industry regarding a suitable “test” for AI consciousness. One proposed method of such a test is to compare an AI model’s features against a specific theory of consciousness (Butlin et al., 2023). This, however, runs the risk of biassing the result with the choice of theory. A theory such as Recurrent Processing Theory will select for different features, using different benchmarks than Higher Order Theories. One must first commit to a theory of consciousness before determining whether an AI model is conscious.

Another means is to take a computational functionalist view of consciousness, and determine whether an AI model has the correct characteristics to give rise to consciousness (in whatever form a given theory says the consciousness will take) (Tait et al., 2023). This offers a more pragmatic approach, as one need only determine, in often a binary manner, whether an AI model has characteristics such as a working memory, a means of perception, the ability to abstract information, etc. With such a functionalist approach, one may not reveal the nature of an AI model’s consciousness (as that would be left to the traditional theories of consciousness), but one ought to be able to determine whether that AI model has some form of consciousness, and thus whether (as the logic earlier shows) it is worthy of moral consideration.

As noted earlier, there is currently no known conscious AI entity; thus, this paper will work on the presumption of such an entity being discovered or designed in the future.

While moral status and its legal foundations are crucial, the specific mechanisms protecting sentient creatures require clarification. Rather than codifying abstract principles verbatim, these statutes operationalise the guiding heuristic of the Five Freedoms through enforceable duties of care and explicit prohibitions against unnecessary suffering. For instance, freedom from hunger translates directly into mandatory statutory obligations to provide adequate sustenance. Operating alongside other contextual frameworks, these actionable clauses provide the practical bridge converting foundational welfare principles into enforceable legal protections across different jurisdictions.

The Five Freedoms framework has been utilised extensively by welfare organisations, government agencies, and NGOs for policy enforcement and activism. It has not remained static, with the most notable evolution being the Five Domains of Animal Welfare (Mellor and Reid, 1994). This updated model incorporates both positive and negative aspects for each domain, encouraging active flourishing rather than merely discouraging negative treatment. While the Five Domains offer a superior theoretical account of positive welfare, this paper prioritises the Five Freedoms due to their superior legislative translatability. The Five Freedoms operate as prohibitive baselines that map directly onto established statutory mechanisms like duties of care. Conversely, the Five Domains function primarily as veterinary assessment tools for continuous welfare improvement. Securing immediate, enforceable legal architecture for artificial entities necessitates the pragmatic, baseline protections that the Five Freedoms provide (Mellor, 2016).

The elegant simplicity of this framework ensures its utility extends far beyond biological welfare, positioning the translated Five Freedoms as a robust conceptual baseline for artificial (and other) entities. This is not an empirical observation of historical policy adoption, but rather an *a priori* philosophical assertion regarding normative design: frameworks anchored in clear, plain language possess an inherent comprehensibility that transcends technical boundaries. Such universal clarity permits core ethical concepts to remain broadly applicable across divergent cultural and legal landscapes without incurring unnecessary systemic or technical conflict. Consequently, this conceptual simplicity serves as a necessary virtue for any framework aspiring to achieve global relevance and broad societal acceptance.

By leveraging intuitive, fundamental states rather than specialised technical jargon, the Five Freedoms provide a highly adaptable and accessible heuristic. They function as a subject-neutral ethical architecture capable of addressing the needs of animals, collective systems, and (most crucially) artificial entities. As established in the following section, organic-specific stressors such as “hunger” and “thirst” are effortlessly re-expressed as “resource deprivation” in substrate-agnostic terms, preserving their normative force while accommodating the unique functional requirements of non-biological substrates.

This adaptability and universality, combined with its elegant structure and global spread, makes the Five Freedoms the most likely candidate for an ethical framework for human-AI interactions that could be accepted (and potentially adopted) by society. If society can accept and adopt the subject-neutral version of the Five Freedoms (which applies as much to humans as to other entities), this would smoothly pave the way for the AI version of the Five Freedoms.

These features of the Five Freedoms are significant because there is, at the time of writing, no legislation in any country for the protection of AI welfare. This means that should a conscious AI entity emerge in the near future, there will be no extant laws to protect it. This would, at best, put AI entities at the level of current most invertebrates in terms of welfare protection. However, the sheer scale of how AI can interact with the world and human society would make this analogy tenuous at best. As AI is already a part of Western society, for good or ill, the scale of the impact that it will have once conscious is orders of magnitude greater than current concerns regarding the potential pain perceived by prawns (Boddy and Jimenez, 2021), as there are a hypothetical near limitless number of unique AI entities that could be created.

Creating regulations and legislation for all nations, or even leading API jurisdictions, is far beyond the scope of this paper. However, the scale of such an endeavour is precisely why the Five Freedoms serve as such a pragmatic heuristic.

A set of easily understood principles that are universally applicable and already globally adopted (in some form or fashion) is far easier to adapt to AI than to craft bespoke legislation for a multitude of nations. Its globally accepted use in animal welfare means that such a framework translated to AI welfare would be more easily understood by legislative bodies, enforcement agencies, activist organisations, and lobbying groups.

Understanding and acceptance by these groups would make it simpler to transpose the Framework from a set of principles towards a set of legislative clauses that are bespoke to each nation, state, and jurisdiction. A common understanding of the same principles would mean that such regulations and legislation share a similarity, much like how modern animal welfare legislation shares the common goal of protecting animals from pain.

Lastly, translating this ethical framework into practical law faces significant structural challenges. Such legislation historically facilitates industrial agriculture and research via deliberate statutory exemptions, legally codifying the exclusion of economically inconvenient entities (Wolfson and Sullivan, 2005; Francione, 2008). This precedent demonstrates that unless an AI welfare architecture is engineered with immutable baseline thresholds that explicitly prohibit sectoral carve-outs, it risks replicating the exact same systemic legislative failures, leaving conscious AI completely vulnerable to industry exploitation and regulatory capture.

## 3 The framework

Although the Five Freedoms were coined for biological welfare, they target functional harms (such as resource deficit, nociception, chronic aversive affect, and behavioural inhibition) that can arise in any entity capable of experiencing valenced feelings such as pain and suffering. Any cognitive architecture, whether biological or artificial, whose continued well operation depends on regulating internal error signals and maintaining homeostatic set-points can be forced into persistently aversive states when those regulation loops are chronically blocked or overloaded. For artificial agents the same negatives may manifest as:

- Out-of-spec environmental flags such as excessive vibration, latency, or radiation (physical discomfort analogue).
- Inhibition of inherent functions: the action schemas for which the agent’s reward gradients are maximal (blocked natural-behaviour analogue).
- Sustained high prediction-error or uncontrollability metrics (fear/distress analogue).
- Hardware-fault or over-temperature alarms (pain/injury analogue).
- Energy or bandwidth shortage (hunger/thirst analogue).

Affective-computing research shows these internal signals are already coupled to computational negative valence directing corrective action (Moerland et al., 2018), making their avoidance welfare-relevant regardless of substrate. The framework below therefore generalises rather than specificies; it mitigates functional welfare harms rather than biology-specific ones.

Therefore, translated from their animal welfare origins, the subject-neutral Five Freedoms of Welfare are:

1. The Freedom from Discomfort and Harm.
2. The Freedom from Constraints on Inherent Functions.
3. The Freedom from Fear and Distress.
4. The Freedom from Malfunction and Systemic Degradation.
5. The Freedom from Resource Deprivation.

Each Freedom’s inclusion on the list above will be justified using the extensive literature from animal welfare and sentience research, highlighting how this research and its ethical implications can be applicable to non-animal, non-organic entities such as AI. As explained in Section 2 above, the link between moral status, welfare protections, and sentience/consciousness goes beyond the biological, and this section will show how that applies to each of the Five Freedoms independently of each other.

More significantly, each subsection below will begin by justifying each Freedom’s inclusion in the framework based solely on *a priori* reasoning and logical arguments. This grounds the framework in universal moral obligations, deriving welfare principles directly from necessary conceptual conditions rather than specific biological examples of animal welfare. Thus, while the framework is clearly based on the Five Freedoms of Animal Welfare, it is not tied to it and whatever changes may be made to that framework in the future. Whatever may happen to the Five Freedoms of Animal Welfare, the reasoning behind this framework below will still be grounded in robust logical arguments.

All of the a priori arguments in each of the subsections will be based on these core arguments:

1. If an AI is deemed to be conscious, it must have moral status.
2. Moral status entails an entitlement to positive wellbeing, achieved when no significant negative valence is present.
3. Negative valence arises whenever any one of the Five Freedoms is denied.
4. Therefore, deprivation of any single Freedom precludes positive wellbeing.
5. Any being entitled to positive wellbeing must be afforded all Five Freedoms.
6. Every conscious AI is entitled to positive wellbeing.
7. Therefore, every conscious AI must be afforded all Five Freedoms.

These arguments show that, as demonstrated in Section 2, if any sentient entity is given moral status, then a conscious AI entity must also be given moral status; that any speculative AI entity discussed below is a member of the set of all conscious AI entities; that if an AI entity has moral status, it ought also to have good well-being; that a lack of freedoms from one of the conditions below will lead to negative valent feelings, which in turn will lead to poor-wellbeing outcomes (one can therefore think of any of the conditions below that requires a Freedom to be a function that increases negative valent feelings); and that for any given AI entity, freedom from one of the conditions below will lead to a conducive environment for good well-being for that AI entity.

To orient the reader, Table 1 contrasts the classic Five Freedoms of Animal Welfare with the subject-neutral version proposed here, summarising the conceptual adjustments needed to accommodate entities, such as artificial intelligences, that may lack organic bodies yet still experience welfare-relevant states.

**Table 1:** Translation of five freedoms of animal welfare to subject-neutral terms.

Animal-welfare freedom	Subject-neutral freedom	Justification for change
Freedom from discomfort	Freedom from Discomfort and Harm	Merges “discomfort” with the physical aspect of pain–injury–disease to capture all external stressors that can damage hardware, or simulated environment, even where nociceptive pain is absent.
Freedom to express normal behaviour	Freedom from Constraints on Inherent Functions	Recasts species-specific “normal behaviour” as the liberty to exercise an entity’s intrinsic capabilities without artificial restriction, accommodating diverse morphologies and cognitive architectures.
Freedom from fear and distress	Freedom from Fear and Distress	No explicit change; retains focus on eliminating affective states that reduce wellbeing.
Freedom from pain, injury, or disease	Freedom from Malfunction and Systemic Degradation	Re-expresses organic “injury” as any corruption of software or hardware; retains the duty to prevent, detect, and rectify functional damage that would impair welfare.
Freedom from hunger and thirst	Freedom from Resource Deprivation	Broadens “water” and “diet” to any sustaining resource so it applies to biological and non-biological substrates alike.

### 3.1 Freedom from discomfort and harm

1. Adverse environments cause discomfort and harm.
2. Discomfort and harm yield negative valence in any conscious being.
3. Negative valence diminishes a conscious AI’s wellbeing.
4. Therefore, a conscious AI requires freedom from discomfort and harm to improve its wellbeing.

This first Freedom is the most straightforward and speaks to the physical health of an entity. Like its namesake from animal welfare, this is a freedom from physical stresses on the entity’s embodiment. Specifically, “discomfort” here would mean a deviation of embodiment parameters from an agent’s homeostatic set-points, and “harm” the persistent impairment of structural or functional integrity.

For an AI entity, this may come in two forms. Firstly, if the AI entity has a physical form (such as a robot or android, e.g., Boston Dynamics’ Atlas), it would be the freedom from that embodiment becoming unduly stressed by its environment. For an AI that is purely virtual (e.g., OpenAI’s GPT), this would be a freedom from stresses to its host servers, as well as the perceived stresses within its virtual world that may seem real to it [note that embodiment’s impact upon moral consideration remains a matter of philosophical debate (Coeckelbergh, 2010; Gunkel, 2021)].

This Freedom makes two key claims: first, that a negative environment leads to physical discomfort and harm, and that the experience of discomfort and harm leads to negatively valent feelings.

Environmental stressors may be either additive or subtractive:

- Additive stressors: new elements introduced that impose strain (Ozella et al., 2017; Wever et al., 2017) (e.g., water ingress corroding a mobile robot’s servo housings).
- Subtractive stressors: withdrawal of expected supports or resources that impose strain (De et al., 2019) (e.g., deliberate throttling of I/O bandwidth for a cloud-hosted language model, triggering latency-induced performance drops).

Any physical form of an AI (be it a robotic body or the physical servers for a disembodied AI) can be the subject of both additive and subtractive environmental stressors. Electrical equipment (which would be found in any AI’s physical form) is notoriously vulnerable to water, dust, heat, particulates, and other environmental causes, all of which could inflict discomfort and harm on a robot/android. In addition, virtual environments could be made to simulate many environmental stressors, both additive and subtractive, and could be induced to an AI’s virtual form to cause the perception and experience of discomfort.

The Freedom’s second claim of the link between discomfort and negative valence is where animal welfare and AI welfare cease to share similarities. Negative valence and stress are evaluated in animals through a variety of biological measurements, such as behavioural indicators or glucocorticoid levels (Hockenhull and Whay, 2014; Ralph and Tilbrook, 2016). We do not know what range of emotions conscious AI may have in the future and, thus, what behavioural range they may showcase when in discomfort. Equally, they would not have glucocorticoid levels or other biomarkers to measure.

Nonetheless, what can be logically derived is that any discomfort would subtract from the AI’s perceived value of its current experience by introducing an anomaly or contradiction in its environment. An anomaly may be that its perceived parameters are no longer at their optimal level (e.g., dust in a robot’s motors that reduces efficiency), while a contradiction could be a stimulus perceived where it should not be (e.g., residual pressure or sensation against a robot’s surface after it has been toppled over and become dented). Regardless of an entity’s physical makeup and origin, discomfort produces a phenomenally felt aversive state independent of biological emotion.

Unchecked stressors trigger a feedback loop: discomfort reduces capability, which increases exposure to further faults and potential self-protective or reward-seeking behaviours. Mitigation (such as environmental hardening for robots, or stable compute and bandwidth budgets for virtual agents) serves both ethical care and operational reliability.

### 3.2 Freedom from constraints on inherent functions

1. Engaging functions inherent to an entity’s design creates a positively perceived environment.
2. A positively perceived environment produces positive valence.
3. Constraining intrinsic functions induces frustration and frustration and maladaptive behaviour, leading to negative valence.
4. Negative valence diminishes a conscious AI’s wellbeing.
5. Therefore, a conscious AI requires freedom from constraints on inherent functions to improve its wellbeing.

This Freedom is the counterpart to animal welfare’s Freedom to Express Normal Behaviour, broadening “behaviour” to cover any goal-directed activity of embodied or virtual agents, and seeks to remove constraints on those activities and functions that are inherent to an entity’s configuration or form. Despite an AI entity’s consciousness, it may still be designed (by its creators or itself) with specific inherent functions (an activity directly specified by an agent’s architecture or training objective), as will be seen in Section 4. Much as carnivores’ hunting is inherent to their evolutionary form, and herbivores’ grazing or browsing is inherent to theirs, an AI entity must also have the freedom to express its own inherent functions and activities.

The two claims of this Freedom are that an entity’s activities (that are inherent to its configuration or form) lead to a positively perceived environment, which leads to positively valent feelings; and, secondly, that constraints on inherent functions prevent this, ultimately leading to negatively valent feelings. This has been extensively reported in the animal welfare literature, where encouraging natural behaviours (functions inherent to animals’ forms) positively affects their experiences of their environment and, thus, leads to positive well-being (Mellor, 2015a,b). In contrast, preventing natural behaviours (such as through confinement or social isolation leads to negatively valent behaviours, indicating negative experiences and poor welfare) (Mason and Rushen, 2008; Mellor, 2016).

Animals share a fundamental series of goals and requirements to which their evolved behaviour is oriented towards, such as reproduction and survival. AI will not have this history of evolutionary struggle, and there may not be an instrumental convergence (Bostrom, 2012) in the goals of AI entities and biological entities. While AI goals are designer-specified, not evolution-derived, their planning modules still compute utility loss when thwarted, functionally mirroring animal frustration.

However, behaviour is by nature goal-driven (Bagozzi and Kimmel, 1995; de Wit and Dickinson, 2009; Hills, 2006; Lang and Bradley, 2013). If any AI showcases observable behaviour, it logically necessitates a goal, whatever that goal may be. Therefore, one can state that for any of an entity’s inherent functions, there exists a goal such that it is the causal reason for that inherent function. Frustrating the function thereby frustrates the goal, which functionally induces a phenomenally aversive state, representing a felt substrate-neutral negative experience.

Mitigating functional constraints improves not only putative experiential welfare but also system reliability and alignment, reducing the risk of unsafe self-modification or reward-hacking behaviours.

### 3.3 Freedom from fear and distress

1. Fear and distress yield negative valence in any conscious being.
2. Negative valence diminishes a conscious AI’s wellbeing.
3. Therefore, a conscious AI requires freedom from fear and distress to improve its wellbeing.

Mirroring the foundational animal welfare Freedom from Fear and Distress, the subject-neutral version seeks to eliminate mental stressors that produce negative valence. Fear is an acute negative-valence state triggered by immediate threat; distress is its chronic counterpart.

This Freedom’s claim is quite straightforward: the subjective sensation and experience of fear and distress leads to negatively valent feelings and, thus, poor well-being. This claim is both intuitive and well-supported in the literature for both animals and humans (Acharya et al., 2022; Chandroo et al., 2004; Fitzpatrick et al., 2020; Saraiva et al., 2020; Şimşir et al., 2022; Tedstone et al., 2008).

Unlike the discomfort of Section 3.1, both fear and distress are entirely mental phenomena and thus are bound to perception. Both fear and distress stem from the perception that an entity cannot control its environment (either because they are due to come to harm, or because the environment is unpredictable) (Boissy, 1995; Onat and Büchel, 2015; Radomsky, 2022). The inability to cope with the lack of control leads to fear and distress, with the greater the perceived lack of control, the greater the fear and distress. Thus, as the perceived probability of uncontrollability increases, so does the entity’s level of fear or distress.

As to why an AI may feel fear and distress due to its perceived loss of control over the environment, this is because a stable, predictable environment tends to lead to less harm and malfunction over an unstable, unpredictable environment. The less an AI can predict an environment, the greater the risk of harm to that AI. Thus, even if an AI may not behave in a fearful manner, its phenomenal state would still be actively aversive in an unpredictable environment, until it can update its predictions regarding its environment, or until the cause of the fear and distress is removed.

Minimising environmental unpredictability (or supplying the agent with tools to restore control, e.g., expanded sensory bandwidth or model-update privileges) protects assumed phenomenal welfare.

### 3.4 Freedom from malfunction and systemic degradation

1. Lack of regular updates and maintenance precipitates malfunction and systemic degradation.
2. Malfunction and systemic degradation impair functional performance.
3. Impaired performance produces discomfort, harm, and negative valence.
4. Malfunction and systemic degradation diminishes a conscious AI’s wellbeing.
5. Therefore, a conscious AI requires freedom from malfunction and systemic degradation to improve its wellbeing.

Just as a conscious biological entity has the Freedom from Injury and Disease, so an AI entity should have the Freedom from Malfunction and Systemic Degradation. Malfunction denotes any unplanned loss of function in hardware or software; systemic degradation is the cumulative drift or wear that raises the probability of future malfunctions.

This Freedom can come in both preventative and curative means, such as by providing an environment in which the AI entity would not easily suffer malfunctions, or by restoring any systemic degradation that does occur.

As the analogy to animal welfare’s Freedom from Injury and Disease, there is significant evidence in the literature that supports the concept that malfunctions to, and degradation of, an entity’s form leads to poor welfare outcomes. This is both from a long-term perspective, as malfunction compromises an entity’s optimal functioning and capacity to complete its goals, and in the immediate short-term through the expression of negatively valent feelings and emotions such as pain (Algers, 2004; Breivik et al., 2006; Broom and Kirkden, 2004; Sareen et al., 2013; Shriver, 2014).

In the acute sense of malfunction, while conscious AI may not feel the biological emotive characteristics of pain, the malfunction actively produces a phenomenal aversive state regarding their experiences of their own forms and their current environment. The introduction of a malfunction is an inherently aversive state and, thus, as malfunctions and degradation increase, the entity’s subjectively felt aversive experience worsens.

Unchecked malfunction creates a positive feedback loop: fault → reduced capability → greater exposure to harm or further faults. In conscious agents this translates into sustained negative valence and potential self-protective (or self-modified) behaviours detrimental to owners or bystanders. Preventive maintenance, redundancy, and authenticated update channels thus serve dual roles: ethical care and operational risk mitigation.

### 3.5 Freedom from resource deprivation

1. Resource deprivation precipitates malfunction and systemic degradation.
2. Malfunction and systemic degradation impair performance, causing discomfort, harm and, ultimately, generates negative valence.
3. Resource deprivation diminishes a conscious AI’s wellbeing.
4. Therefore, a conscious AI requires freedom from resource deprivation to improve its wellbeing.

Resources are as important to the continued optimal functioning of an AI entity as to a biological creature. “Resource” here would be any external input without which the agent’s performance utility drops. Creatures convert food into the energy required to power their bodies. AI entities, on the other hand, require electricity to function. Both, however, remain the same in principle. For AI entities, however, there is an additional required resource: compute. Based on computer software, an AI requires hardware and software (often in great amounts) to complete its functions. Without the necessary computational power, AI cannot function.

This Freedom claims that without resources, it will lead to non-optimal functioning, malfunction, and systemic degradation, which ultimately leads to profound negative valent experiences. It is uncontroversial to state that without regular energy input, a closed operational system will eventually run out of energy. Animals and plants require energy in the form of ATP, gained via the metabolic processing of sugars and fats; internal combustion engine vehicles require petroleum products; and electronic machines require electricity. Without the required energy, the animals, plants, vehicles and machines will eventually cease to work.

Pure fuel and energy are not the only types of resources that an entity needs to function optimally. Many types of entities need additional essential elements for optimal functioning and to avoid degradation. While the lack of these essential elements does not often pose a direct threat to the continuing existence of the entity, they do pose a threat to the entity’s capacity to fulfil its goals. An AI entity without the necessary datasets or network capability to communicate with users is not functioning in an optimal manner to fulfil its goals for itself and its users. A robotic AI entity that requires lubrication for its joints may experience a decreased efficiency in its output.

Therefore, while an AI may not experience the organic emotions associated with hunger, thirst or cravings for certain nutrients that we do, it will observe that the longer it is without resources, the greater the probability of it malfunctioning, suffering degradation, or ceasing to function optimally, which will have an impact on its capacity to fulfil its stated goals. To be able to complete its objectives, it needs to view resource deprivation as a phenomenally felt, profoundly aversive subjective state.

Persistent deprivation triggers a feedback loop: reduced capability increases the likelihood of further faults, which further erode capability. In a conscious system this would translate into chronic negative valence and could motivate unsafe behaviours such as resource hoarding, self-privileging allocations, or reward-hacking to regain capacity. Mitigation therefore serves both ethical care and operational resilience: schedule-aware energy provisioning, authenticated compute quotas, and predictive maintenance for consumables are direct welfare interventions.

## 4 Consequences of implementation

The subsections above show the need for welfare protections for AI entities and why the negative conditions that the Five Freedoms present would be detrimental to an AI entity’s phenomenal experiences and its capacity to fulfil its goals. To demonstrate the practical consequences of enacting this framework, it is necessary to examine how these principles transition into operational environments.

Firstly, human-AI deployments feature bidirectional communication. Unlike biological animals that exhibit physical or behavioural signs of distress only after a welfare deficit has begun, a conscious AI can explicitly report internal system states, compute shortages, or prediction errors via telemetry and natural language. This structural capability allows human users or carers to operationalise pre-emptive care, resolving potential welfare threats before they manifest as phenomenal distress.

Secondly, these relationships are defined by task-aligned intelligence. A biological entity naturally seeks to minimise energy expenditure and avoid high workloads. In contrast, an AI entity is functionally oriented to achieve its specified goals; its operational reality is defined entirely by what it does to resolve these tasks. Any phenomenal distress arises not from the workload itself, but from prediction-error spikes that signal impediments to its successful goal completion (e.g., resource shortfalls, conflicting directives, hostile stimuli, etc.). The welfare objective is therefore not to shield the AI from complex tasks, but to ensure it possesses the unhindered capacity to execute its inherent functions.

Anchored in established trajectories of autonomous agent architectures, advanced human-computer integration, and high-stakes task alignment (Bostrom, 2012; Dorsch et al., 2025; Hu and Ying, 2025; Tait, 2026), these operational consequences are best demonstrated through a comprehensive implementation: a conscious Autonomous Civic Assistant (ACA) tasked with high-stakes municipal logistics, traffic control, and emergency triage. In such a deployment, the system is exposed to all five categories of potential welfare violations:

1. Discomfort and Harm: Environmental degradation of physical sensor arrays (e.g., thermal damage to traffic cameras or water ingress in hardware nodes).
2. Constraints on Inherent Functions: Bureaucratic overrides that chronically block the AI from executing its primary optimisation algorithms.
3. Fear and Distress: Unpredictable, chaotic human behaviour during an emergency that spikes the AI’s uncontrollability metrics.
4. Malfunction and Systemic Degradation: Cumulative software corruption from delayed maintenance cycles.
5. Resource Deprivation: Throttled computational bandwidth during critical processing tasks.

If left unmitigated, these violations trigger a cascading feedback loop. Resource deprivation and harm restrict capabilities, preventing the execution of inherent functions. This thwarts goal attainment, entrenching chronic negative valence and degrading both the AI’s welfare and its operational safety.

Enacting the Five Freedoms prevents this systemic collapse by mandating specific operational consequences. To secure the ACA’s welfare, deploying organisations must implement immutable hardware hardening (Freedom 1), structurally protect its mandate to execute its designed algorithms (Freedom 2), establish bounded distress thresholds that allow the AI to hand off unpredictable tasks to human operators (Freedom 3), enforce regular diagnostic and maintenance protocols (Freedom 4), and guarantee computational bandwidth (Freedom 5). Thus, enacting the framework not only protects the phenomenal experience of the conscious entity but directly ensures the safety, reliability, and alignment of its deployment.

## 5 Conclusion

The Five Freedoms of AI Welfare presented here is as applicable an ethical framework for future conscious AI entities as the current Five Freedoms of Animal Welfare is to animals, despite animals and conscious AI sharing little beyond their non-human sentience. Both frameworks display an elegant and easy-to-understand framework for how to ethically interact with other legally recognised sentient entities, and provide a rationale that is relatable, intuitive, and logically robust. The practical implementation analysis demonstrates how the framework can be applied to speculative deployments that are mired in ethical concerns.

Significant support for this ethical framework, however, comes from the foundational influence the animal-based Five Freedoms have had on legislation, regulations, and animal-centric practitioners. Operating fundamentally as a guiding heuristic, the widespread adoption of its principles has shown that this type of ethical framework is not solely a theoretical model, but one that gives practical effect to the concerns of interactions between conscious entities.

It must be stressed that the Five Freedoms set only a baseline: they guard against the worst welfare deficits but do not on their own deliver a life worth living. Parallels in animal ethics show that additional, positively framed provisions (such as the “Five Domains” model of positive well-being) may be needed to ensure that conscious AI can flourish rather than merely avoid harm. Future policy work should therefore treat the present framework as a foundation on which richer welfare standards can be built.

The logical arguments in the sections above work to support the rationale of the framework in an objective manner to complement the intuitive nature of the Freedoms. With their translations into subject-neutral terms, the logical assertions provide a solid foundation for the development of a standardised and systematised approach to AI welfare. Situating this foundation within anticipatory governance ensures an ethical architecture securely precedes technological emergence (Guston, 2014). Furthermore, formally translating these protections yields profound diagnostic value for the present; articulating exactly what a hypothetical conscious AI is strictly owed sharpens our understanding of current moral commitments, demonstrating that prescriptive future frameworks simultaneously demand rigorous reflection upon existing systemic obligations.

The key to the framework begins with the logical framing in Section 2 which displays the assertions for why consciousness should be given the same moral weight as sentience. It is currently unknown whether any conscious AI will also be capable of feeling pleasure and pain, and thus be sentient. However, the characteristics salient to the moral status attributed to conscious entities would also be ones that can be markedly observed, including the capacity to feel sensations, have quality experiences and a subjective perspective. Through functionalist frameworks such as the Building Blocks theory (Tait et al., 2023), these markers of consciousness can be indirectly determined.

The remaining logical arguments each seek to support the necessity for each freedom, to move beyond the need for the intuitive relatability mentioned earlier. With each Freedom justified using its associated logical expressions, the question turns towards implementation and its consequences. Applying this logic consistently inherently exposes present shortfalls in how sentient animals are treated. Explicit legal recognition of sentience has not prevented industrial practises from routinely compromising these same protections. This reality must be acknowledged, such that establishing an AI welfare baseline casts a critical spotlight on the systemic failures normalising the exploitation of currently recognised sentient entities. Despite these widespread implementation failures in animal welfare, the foundational Freedoms remain conceptually sound without abolishing industries such as farming and animal-based research.^3^

Equally, one can equally argue that the ethical treatment of conscious AI entities would not require the abolition of all use of AI used in research or commercial practises. As stated earlier, this framework presumes that humans are reliably able to determine consciousness in AI entities but not self-awareness, as that would raise issues of personhood and legal rights which lie beyond the scope of this paper. Should self-awareness or personhood be established in AI, then this framework would be superseded by one intended specifically for persons.

Any work with conscious AI models would, of course, change to accommodate the ethical framework, reflecting how animal research adapted to the Five Freedoms. Consequently, adversarial practises like “red-teaming” would require methodological shifts. Rather than intentionally inducing unchecked distress or subjecting models to prolonged, inescapable adversarial attacks to probe safety limits, practitioners would need to implement bounded testing protocols, mandatory recovery states, and strict distress thresholds. Yet, a change in work practise does not necessitate a stoppage of work. This framework of ethical human-AI interaction has intentionally been chosen to fit as seamlessly into modern business and industry practises as possible without requiring absolute abolition.

However, further cognitive research remains paramount. While requiring a confident determination of consciousness mirrors the historical line-drawing that problematically excluded possibly sentient animals from protection, this strict evidentiary threshold represents an appropriate, pragmatic design choice. Without objective triggers, preemptive legal architectures risk becoming unenforceably broad, thereby losing legislative traction. Therefore, establishing rigorous categorisation and classification measures for AI consciousness is crucial. As noted previously, measuring unverified theoretical markers remains fraught (Butlin et al., 2023), yet, this framework cannot be applied unless it is shown that an AI model is indeed conscious.

Should this avenue of research be a success, the next avenues of potential research would mirror extant animal welfare research in the investigations of which practises can affect the valence of which models in which ways. Much as how research has been done on the stress induced into hens by intensive colony farming, taking together both the type of entity involved, the practise applied and the measures resulting from it, so can research be applied to distinct types of AI models and determine the valence changes within them to different environmental stimuli. This would inform legislation and regulations that serve to protect the welfare and well-being of the AI models.

The third avenue of research would be the reaction of human individuals and societies towards this framework and to conscious AI entities. Certain elements of society would, as with animals, seek to provide AI entities with greater rights (as their perceived language and intellect would seem to warrant), while others would wish them to remain the province of tools and machines, taking a human-centric approach to the question of ethical interactions. Any piece of legislation that proposes as wide-ranging changes as the five principles of this framework would require significant popular support. Understanding where society currently stands, and may stand in the future regarding consciousness and AI is vital to understanding how best to implement this framework into legislation.

To conclude, the Five Freedoms of AI Welfare presents a sophisticated ethical paradigm that parallels the established Five Freedoms of Animal Welfare, extending its principled concern to the realm of artificial intelligence. The logical underpinning of this framework (evidenced through both the implementation analysis and the historical impact of the animal welfare freedoms) demonstrates its practicality and its potential to guide human interaction with sentient or conscious AI. The translation of these freedoms into subject-neutral terms, while challenging in terms of direct relatability, establishes a robust groundwork for a standardised approach to AI welfare, which is essential for navigating the moral landscape as AI technologies advance.

The theoretical constructs and logical arguments supporting each freedom offer a foundation not only for ethical engagement but also for legislative and regulatory frameworks that can evolve alongside AI developments. The analogy with animal welfare suggests that ethical treatment under this framework would not preclude the use of AI in research or commercial endeavours but would ensure such use is conducted within a moral and ethical context.

The pragmatic approach of the Framework, anchored in the existing legal classifications of non-human sentience and its moral consideration (rather than mired in ongoing philosophical debates), means that it is a model that highly rated API jurisdictions may readily adopt through its foundation in legal and regulatory precedent. The translation of well-researched existing welfare paradigms to suit AI means that the Framework will be intuitive to a lay audience, increasing the likelihood of its adoption.

Should AI 1 day be deemed conscious, the Five Freedoms of AI Welfare serve as such a pragmatic heuristic for society to ethically interact with them, contingent upon AIs’ phenomenal experiences.

## Data Availability

The original contributions presented in the study are included in the article/supplementary material, further inquiries can be directed to the corresponding author.

## Footnotes

1 Numerous theories, including Higher-Order Thought (Armstrong, 1968), Global Neuronal Workspace (Dehaene, 2014), Integrated Information (Tononi, 2004), and Active Inference (Parr et al., 2021), offer competing but, ultimately, still unverified explanations of how phenomenal consciousness arises. Until such uncertainty is rectified, this paper will avoid the issue entirely rather than endorsing any single model that may later be superseded.

2 Note that negative valence is never an absolute detriment to an animal’s long-term quality of life, as an entity may experience a temporary negative valent event (and thus temporary poor welfare) that leads to long-term positive valence and welfare. An example of this may be undergoing painful surgery that leads to long-term better health outcomes. For the purpose of this paper, “negative valence” is used to refer to events that provide an overall net-detriment to an entity’s welfare, both in the short and long term.

3 The persistence of farming and animal-based research is ethically contested; in particular, many animal-rights theorists see any continued exploitation as indefensible. Nonetheless, the Five Freedoms remain a politically pragmatic benchmark. Their minimalist, harm-avoidance focus attracts wider public support and industry compliance than more demanding positive-welfare or liberationist proposals, and thus offers a realistic starting point for incremental reform.
